# Supplementary material for: Fine-scale family structure shapes influenza transmission risk in households: Insights from primary schools in Matsumoto city, 2014/15
Source: PLoS Comput Biol. 2019 Dec 26;15(12):e1007589. doi: 10.1371/journal.pcbi.1007589 (PMC6959609; doi:10.1371/journal.pcbi.1007589)
Supplement: Attachment — Submitted filename: comments.pdf [file pcbi.1007589.s002.pdf]

In this manuscript, the authors apply mathematical models to infer age-specific effective contact rates of influenza transmission in household settings, based on a large-scale school and household surveys in Matsumoto, Japan. There are several interesting findings from the study. In particular, the study confirms that school age children are more likely to obtain infections from outside and introduce influenza into the household, as previous studies have been suggested. The authors also identify significant lower risk of cross-generation influenza transmissions except the "mother-child" contact and a significant higher risk of "elderly-elderly" transmission when compared to other age groups. These findings illuminate the transmission pathways within the household and shied lights on their implications on intervention strategies. The study also explores the "density-dependent" vs. "frequency-dependent" mixing assumptions. The fitting results favors the mid-way hybrid of two style of mixing, indicating both contact strength and contact number (household size) matter. The experiment is expertly designed; the analysis is carefully conducted with sound statistical analysis as well as adequate sensitivity analysis; the manuscript is clearly written. I'd like to recommend it for publication once the following suggestions are addressed:

- It's helpful if the authors could provide some background of influenza circulation in the general population in Matsumoto during 2014/2015. Was it a predominately influenza A or influenza B season? What's the proportion of H1N1 vs H3N2? How severe the epidemic season when compared to other years in Japan? What was the vaccine coverage/effectiveness in the population? Theses information are relevant as a reference because the transmission dynamics of different influenza subtypes/strains are not the same and may have divergent impacts on different age groups.
- I suggest the author to move line 208-221 before 195-207. It's better that c_{kl} is defined ahead of C_k. Otherwise it's difficult to follow the reasoning of mixing assumptions.
- Was the estimation of gamma (mixing parameter) also robust in the sensitivity analysis? The estimation of gamma ~ 0.5 is an interesting finding and shall also be tested in the sensitivity analysis.
