# Supplementary material for: Fine-scale family structure shapes influenza transmission risk in households: Insights from primary schools in Matsumoto city, 2014/15
Source: PLoS Comput Biol. 2019 Dec 26;15(12):e1007589. doi: 10.1371/journal.pcbi.1007589 (PMC6959609; doi:10.1371/journal.pcbi.1007589)
Supplement: Attachment — Submitted filename: pbp_rev1.docx [file pcbi.1007589.s003.docx]

**Point-by-point responses**

Reviewer #1:

In this manuscript, the authors apply mathematical models to infer age-specific effective contact rates of influenza transmission in household settings, based on a large-scale school and household surveys in Matsumoto, Japan. There are several interesting findings from the study. In particular, the study confirms that school age children are more likely to obtain infections from outside and introduce influenza into the household, as previous studies have been suggested. The authors also identify significant lower risk of cross-generation influenza transmissions except the “mother-child” contact and a significant higher risk of “elderly-elderly” transmission when compared to other age groups. These findings illuminate the transmission pathways within the household and shied lights on their implications on intervention strategies. The study also explores the “density-dependent” vs. “frequency-dependent” mixing assumptions. The fitting results favors the mid-way hybrid of two style of mixing, indicating both contact strength and contact number (household size) matter. The experiment is expertly designed; the analysis is carefully conducted with sound statistical analysis as well as adequate sensitivity analysis; the manuscript is clearly written. I’d like to recommend it for publication once the following suggestions are addressed:

• It’s helpful if the authors could provide some background of influenza circulation in the general population in Matsumoto during 2014/2015. Was it a predominately influenza A or influenza B season? What’s the proportion of H1N1 vs H3N2? How severe the epidemic season when compared to other years in Japan? What was the vaccine coverage/effectiveness in the population? Theses information are relevant as a reference because the transmission dynamics of different influenza subtypes/strains are not the same and may have divergent impacts on different age groups.

> We thank the reviewer for important suggestions. Although the city-level virus circulation data was not available as Matsumoto city started reporting surveillance results from the 2015/16 season onwards, the national surveillance data suggested AH3 strain was predominant that year. We mentioned epidemiological backgrounds related to the present study in Lines 116-122 (copied below), and also highlighted that the readers can refer to the original studies for further information.

*The influenza types reported for the student cases during the season were mostly A (95% of those tested positive) (30). The national-level surveillance data suggested that AH3 strain was predominant, accounting for 99% of the type A isolates (31). The vaccination coverage of the students in the dataset was 48%; however, we did not consider vaccination in the present analysis. Further details of the data collection and descriptive epidemiology can be found in the original studies (29,30).*

• I suggest the author to move line 208-221 before 195-207. It’s better that ckl is defined ahead of Ck. Otherwise it’s difficult to follow the reasoning of mixing assumptions.

> We agree that the original manuscript could have been clearer in providing an overview of variable definitions. Since the original lines 208-221 were too detailed to present right after Eq.4, we added a short description of variables c_kl_ and beta in Lines 2199-220:

*The contact intensity ckl represents the (hypothetical) number of household contacts between type k and l, and β is the transmissibility coefficient.*

We believe that now the readers can see the brief definition of each parameter first, and then move on to the details in the following paragraphs.

• Was the estimation of gamma (mixing parameter) also robust in the sensitivity analysis? The estimation of gamma ~ 0.5 is an interesting finding and shall also be tested in the sensitivity analysis

> We found that the value of gamma was also robust in the sensitivity analysis (mostly ~ 0.5, and 0.6 when case censoring was considered(S3iv)). We have noted this in the updated supplement (S3, “Result of the sensitivity analysis”):

*The exponent parameter γ was stable throughout the sensitivity analysis (median within 0.50 ± 0.02), except that it was slightly higher (0.59; CrI: 0.39-0.79) when the case censoring (iv) was considered.*

Reviewer #2: Review of Endo et al

I would like to start by asking whether you may accept my apologies for submitting the review late. I hope I have not inconvenienced the authors or editors too much.
Endo and colleagues have written a thorough analysis of a very impressive dataset, of self-reported influenza infections from ~10 000 households in Matsumoto, a lovely city in Japan nestled in the northern alps. The dataset is to-die-for: 10 000 (/13 000) primary school students’ parents reported the household structure is captured to a fairly high resolution—though duplicate entries may have arisen and there is some difficulty in enumerating siblings—and whether they were infected by influenza over the course of a season. The authors note that Japanese doctors often test for influenza infection with a rapid diagnostic test (which is true, though I have no idea why they do this) which minimises the diagnostic bias that comes from requiring self-reporting. They use these data to fit models that let them distinguish the differential rates of transmission between agent types and to identify that the contact rate lies somewhere between density and frequency dependence. These are interesting findings that advance our knowledge of influenza transmission in households. I have some suggestions for improvement in the presentation of the results that I would recommend the authors consider.
My primary concern is why are the estimated infection risks from outside the household so close to the final attack rates observed empirically? ~20% of students were infected from outside vs 20.5% of students being infected in total. ~16% of siblings were infected from outside vs 18% infected in total. (Etc) This suggests that households are almost wholy irrelevant for influenza transmission, which does not accord with the authors’ own description of the importance of households for transmission or with the findings of other studies. Is the distribution of final attack sizes in their data close to binomially distributed? This should follow from these transmission rates, and would be a good empirical test of validity of the finding and estimates.
Of course the other way odd results could arise would be due to a coding mistake. Have the authors simulated a fake dataset (with substantial within household transmission) and shown that the routine can recover the parameters and hence behaviour used?

> We thank the reviewer for valuable feedback. As shown in Figure 3, the overall risk of infection in children mostly came from outside the household. This was because (a) we estimated they have a much higher risk of infection at schools and (b) they are less likely to be exposed to infectious household members as adults have a lower risk of infection from outside the household. However, this does not mean that within-household transmission is irrelevant for children: once a member of the household is infected, children are still exposed to the additional risk of infection (see, for example, the additional risk caused by an infected student in Fig 5G-H) – The distribution in our dataset was clearly distinct from a null binomial distribution. For example, out of 9,135 pairs of parents (father and mother living together) in our dataset, the proportions of “both uninfected”, “only one infected” and “both infected” were 87%, 10.5% and 2.5%, respectively. If we assume a binomial distribution while keeping the “both uninfected” proportion as observed, we should observe 87%, 12.5% and 0.5%. That is, we observed substantially more “both infected” pairs than we would in the independent situation (p-value<10^-5^), and this is reflected in our (non-zero) estimates of within-household transmission risks. We believe a new figure in the supplementary materials (Fig S3: The observed and predicted final attack size distributions) also helps to show the distribution was not binomial.

*(Supplementary materials: “Model fit” section) We also compared the predicted and observed distributions of the final attack size (the total number of household cases during the season) for specific compositions in Figure S3. The observed distribution was right-skewed compared to the binomial scenario, where within-household transmission is not present and individuals are assumed to be exposed to the external risk of infection only.*

Also, although we did not explicitly use simulation, we tested our code by checking its mathematical properties (probabilities should sum up to 1; consistent with the closed-form solutions available for simple cases) and we are confident that it worked in the way we expected. To ensure transparency and reproducibility, we have added the test code to our Github repository (<https://github.com/akira-endo/HHstudy_FluMatsumoto2014-15/blob/master/codetest.r>).

My other main critique is that the authors don’t really demonstrate the validity of their models except through a non-intuitive model fit section in the appendix. This merely compares the proportions of households with a particular configuration in model and data, but doesn’t show the distributions of configurations directly so does not allow the reader to assess whether there are systematic biases as might be caused by inability to capture the correlation structure correctly. Also the way they present these obfuscate the comparison because there is no like-for-like comparison.

Instead I would strongly recommend the authors present final attack size distributions for households of varying sizes (probably summing over configurations within a size) as this will clearly and directly show whether the model and data accord.

> We believe that Figure S2 is important as it allows comparison of the number of occurrence of each specific household composition/outcome in the model and data at one glance, without summarising. Moreover, the empirical KL divergence of 0.05 indicates that the predicted and observed distribution of household outcomes are close enough. However, we do agree that it would be helpful to include an intuitive comparisons of other patterns in the dataset. We have therefore added a new figure comparing final size distributions for different households (Fig S3).

It is great that the authors provide code on github. I would invite them to think also of what kinds of output from their analysis might other researchers want to use as inputs into other papers, and consider whether such output can also be provided? For instance, one would expect that transmission risk parameters as a function of ego and household composition would be very useful for other simulation models: would the authors consider providing this (or other output?) in a user-friendly way?

> We appreciate that the risk of infection given the type of an individual and their household composition would be of particular interest to others, which was the motivation for Figure 5. Although we only included primary and secondary infections here, the overall risk is mostly accounted for by the primary and secondary infections, and therefore should be sufficient to characterise an individual’s risk. To allow others to make use of these outputs, we have uploaded these risks in an RData format on Github so that other researchers can easily reuse them. We also made posterior samples publicly available.

How tenable is the assumption that there is no transmission between households? Clearly it is not in reality true, but how much does ignoring this invalidate the likelihood?

> We acknowledge that transmission between households is not explicitly expressed in our likelihood function. However, such between-household transmission risk is implicitly incorporated in the external risk parameter epsilon. That is, epsilon may be understood as “(infection risk from other households) + (infection risk from other places)”. We did not break down epsilon into such parameters because they would not be identifiable as long as we assume homogeneous mixing between households (which would be inevitable as we do not know which pairs of households were “in contact” with each other). Nevertheless, we believe that the estimates of the external risk of infection as a whole (from schools, workplaces, other households and general community altogether) are still useful as an overall assessment of risk.

Minor comments:

Please clarify equation 1: does epsilon apply ONCE per season or is it applied once each generation of infection? Logically it must be the former, but my interpretation of the equation (which may be wrong!) led me to think that it may be the latter. Please clarify.

> Yes, epsilon applies only once per person, and our equation is also based on that assumption. Please note, although we used recursion in Equation 1, this was only for the computational purpose and the recursion does not represent infection steps. We have modified Lines 172 and 181 to clarify that epsilon is the risk over the whole season.

*We denoted by* ***ε*** *the external risk of infection over the season for each type of individual.*

$S_{k}\left( \boldsymbol{n,\varepsilon} \right)$*, the probability that a type k individual escapes infection from both outside and inside the household throughout the season, is…*

The authors should mention in their ethics statement that the original study was approved by a Japanese IRB.

> We have added a sentence about the local approval (Lines 516-517). Also we have corrected the confusion of the reference numbers.

Table 2 probably could appear in the appendix as it is rather dull compared to the other exciting parts of the paper.

> Although we agree that Table 2 might also be placed in the supplementary materials, we wish to leave it in the main text for the readers’ reference: the notations for household compositions (such as FM-1) are used in some of the main figures and the readers might want to refer to the notation as well as the proportion in the dataset.

Line 132: presumably it was not primary school students but their parents who did the reporting?

> We have edited the sentence to clarify it was not the students who reported. We mentioned in Line 102 that the parents filled in the questionnaires.

Line 153 (and elsewhere): best not to start sentences with lower case letters, even if they are Greek. Can this be reworded to avoid this? Eg The risk of external infection, epsilon, for each type of etc…

> We have modified the sentences to avoid lower case Greek letters.

Line 173: This section may be better before Line 145 as it helps clarify the entries in N and n.

> We reconsidered that this paragraph is more about the dataset and thus have moved it to the “Data source” section (Line 136-148, which is before the original Line 145).

Line 241: Define ESS on first usage. (Currently defined on second usage…)

> We have spelled out the first one instead of the second.

Table 3: clarify why beta is not sampled. Presumably because it is a derived quantity?

> Yes, we indicated this in the table.

Line 299: The issue of not capturing multiple events among siblings should be addressable directly through the likelihood function, using the property exemplified in the following case. If X=(x1,x2,x3,x4) is MNM with parameters n and p=(p1,p2,p3,p4) then X’=(x1,x2,x3+x4) is MNM with parameters n and p’=(p1,p2,p3+p4).

> The difficulty in addressing truncations of siblings here was that both N (household composition) and n (members infected) were truncated when there could have been more than one. That was the reason why we needed rather complex mathematics in S3iv. We clarified that we modified the likelihood to address this issue in Line 304. Also, because of the within-household transmission (i.e. n was not binomial-distributed), our likelihood does not have the reproductive property.

Line 466: Surely it goes without saying that subclinical infections were present!

> We replaced “may have been” with “were probably” to indicate subclinical infections were fairly likely.

The authors could remove a few abbreviations: RDK, WBIC, CPI are scarcely used.

> We decided not to use the abbreviation RDK (and used “rapid diagnostic tests” instead as this seems a more accepted term). We realised CPI need not necessarily mentioned and removed from the text. We wish to keep WBIC since this may be better known than its full form (also it is used throughout the supplement so the readers will see them anyway).

Figure 2: Consider rotating these panels by 90 degrees to allow the labels currently on the x axis to appear horizontally on the y axis. The CIs on panel B come out hard to see in a B&W printer. Consider modifying the colour.
Figure 3+: Ditto, consider rotation to improve readability

> We have modified the colour of Figure 2B. We understand that the rotated labels are not the best to read, but we still wish to show the bars vertically as it would help the readers to get better quantitative impression. We use the consistent orders for labels throughout the figures so hopefully it would not affect the readers too much.

Appendix after equation S1: Were there really no same-sex parents in >10 000 households? Isn’t Matsumoto quite bohemian given the density of artists there?

> We did not find same-sex parents in the dataset (at least not in any way we could identify from the data available). This may be because our samples were limited to families with at least one primary school age child (adoption by same-sex parents has not been easy until recently, although the situation is gradually changing). However, we slightly modified the sentence so that it will not give an impression as if such cases were impossible.

Appendix S3: what is the subscript in line 2 of ascertainment bias?

> The subscript was **n’**>=**n**. We slightly edited the math so that it would look better.

Appendix S3ii: What are children adults? (Sounds like a rival to glay)

> We have corrected the typo (no, not Mr C…)

Finally the authors should give both the main manuscript and the supplement a more careful going over: there are some English lapses in the former and quite a few in the latter. As I understand, the supplement is not copy edited at all, so the authors should be quite careful lest their work appear sloppy.

> The manuscripts have now gone over a grammar check and most errors should have been corrected.
